# Post-Crash First Response by Traffic Police in Nepal: A Feasibility Study

**DOI:** 10.3390/ijerph19148481

**Published:** 2022-07-11

**Authors:** Gary Smart, Amrit Banstola, Raju Raut, Krishna Ghimire, Julie Mytton, Elisha Joshi, Sunil Joshi

**Affiliations:** 1 Faculty of Health and Applied Sciences, University of the West of England, Bristol BS16 1DD, UK; julie.mytton@uwe.ac.uk; 2 Division of Global Public Health, Department of Health Sciences, Brunel University London, London UB8 3PH, UK; amrit.banstola@brunel.ac.uk; 3 Nepal Red Cross Society, First Aid Division, Kathmandu 44614, Nepal; raju.raut@nrcs.org (R.R.); krishna.ghimire@nrcs.org (K.G.); 4 Nepal Injury Research Centre, Kathmandu Medical College, Kathmandu University, Kathmandu 44600, Nepal; ejoshi03@gmail.com (E.J.); sunil.joshi@uwe.ac.uk (S.J.)

**Keywords:** first responders, prehospital care, trauma, traffic police, training

## Abstract

Background: Road traffic injuries are a significant and increasing public health burden in Nepal, but there is no national coverage of regulated and standardized emergency medical service systems. Therefore, this study was designed to develop a first responder trauma training program for the Nepal traffic police and to evaluate the feasibility of its delivery and follow up. Methods: A training needs assessment with traffic-police officers in a single district of Nepal informed the development of a 3-day first-response course which was provided to officers in May 2019. Participants were supplied with a trauma-pack and asked to complete a report form when first-responder skills were used. Knowledge and confidence face-to-face surveys were used before and after training to assess learning, and were repeated at 6 months to assess retention of knowledge. The surveys at 6 months assessed the factors affecting application of first response skills. Results: Most (97%) participants believed giving first-aid was part of their responsibilities and 95% had experience of transporting road crash victims to hospital with a range of injuries. Low levels of first-aid training and variable course content were reported. Knowledge and confidence scores improved post-intervention but were reduced at 6-months. During attendance at 303 road crashes in the 6-months follow-up period, 44% of the participants self-reported using at least one skill from the course; applying them on 92 occasions. Incident report-forms were frequently not completed. Barriers to providing treatment included: the patient already en-route to hospital when police arrived at scene; resistance to providing care from relatives or bystanders; and competing police duties (e.g., traffic management). Conclusions: Delivering a first-response training program for traffic-police in Nepal is feasible. Knowledge was retained and used, and skills were in frequent demand. A study of effectiveness and cost-effectiveness appears warranted to determine if extending the training to other districts can improve outcomes in road traffic injury patients in the absence of formal emergency medical services.

## 1. Introduction

Organized prehospital trauma care has been shown to be effective in reducing death and disability from road traffic injuries (RTI). RTI victims need effective post-crash emergency care systems to provide care at the scene, prompt transport with care on route, followed by facility-based emergency care [1]. A study comparing prehospital mortality rates identified that deaths occurred at higher rates in low and middle-income countries (LMICs) (81%) when compared with highly organized emergency medical services (EMS) in high-income (59%) settings [2].

Road traffic injuries are a significant public-health burden in Nepal, with increasing levels of morbidity and mortality [3]. In 2017, an estimated 7524 deaths (4.11% of all deaths in Nepal) were attributed to transport injuries, making it the 7th leading cause of death in the country [4]. Prehospital care in Nepal is sub-optimal, and with a few notable exceptions, there is a lack of regulated and standardized EMS systems [5,6,7,8]. Most ambulances are poorly equipped with only an oxygen tank and a single driver who may or may not have received basic first-aid training. Most are not crewed by trained emergency medical technicians or paramedics and poorly equipped to manage trauma emergencies [5,6]. Most patients arriving at emergency departments do not arrive by ambulance, but arrive by taxi, private-vehicle, bus or motorbike [5].

The Government of Nepal and the World Health Organization recommend a hub and spoke model of trauma care, and emphasized the need to make Emergency Health services available at all levels including Basic Health Centers and Primary Hospitals. They support the need to establish trauma care centers in strategic areas on major highways; expand ambulance services to all municipalities and provide helicopter EMS to extremely rural areas [9,10].

Following a road traffic collision in Nepal, the Nepal Traffic Police (NTP) are usually the first official response and reliable presence at the scene [6]. Traffic police are trained in simple first aid, however, receive limited training in the management of RTI victims. There is limited research on injuries occurring to residents of Nepal, including road traffic injuries [11] and there is currently no research on the traffic-police officers’ experience of providing emergency care at the crash scene. Therefore, this study was designed to develop a first responder trauma training program for the NTP and to evaluate the feasibility of its delivery and follow up.

## 2. Materials and Methods

The study was conducted in a single district in Nepal. The district has a mixed geographical terrain, ranging from low-land to high hills and it reflects a large range of socio-economic settings found in Nepal, thus results from studies in this district have the potential to be generalized to other similar districts across Nepal.

The study team worked with the Nepal Red Cross Society (NRCS), an established non-profit organization and auxiliary to the government in the humanitarian field. Based in Kathmandu, the NRCS has extensive experience in providing first-aid training for community groups, businesses and government bodies [12].

Our aim was to develop an intervention and evaluate the feasibility of its delivery to traffic police, therefore we identified a district with whom we could work collaboratively, over a defined period. The study participants were all the traffic police officers stationed in the district. Provision of first aid is made explicit in the NTP mission objectives which state that the police should “Render assistance to the public in various stressful conditions such as prompt first aid to accident victims” [13].

The study was divided in to three phases: intervention development, intervention delivery incorporating a pre-test, post-test design, and a 6-month follow-up. All face-to-face verbal surveys were conducted by the same interviewer to ensure reliability.

Ethical approval was obtained from the Ethical Review Board of the Nepal Health Research Council and from the Faculty Research Ethics Committee of the University of the West of England, Bristol, UK, prior to commencement of the study.

### 2.1. Phase 1: Intervention Development

A training needs assessment of all the traffic police officers working in Makwanpur district was conducted. A structured questionnaire (a combination of 19 open and closed questions) was used to conduct face-to-face verbal surveys with individual traffic police officers (Supplementary File S1). The survey explored their prior training in first aid and experience of transporting and/or providing first aid to injured persons. The surveys were conducted in the Nepali language by a Nepalese researcher.

The information from the survey was used to inform the design and content of a bespoke 3-day Trauma First-Responder Course (Supplementary File S2). The curriculum incorporated guidance from two main sources: the International Federation of Red Cross and Red Crescent Societies International First Aid and Resuscitation Guidelines 2016 [14] and the WHO Basic Emergency Care Course 2018 [15].

### 2.2. Phase 2: Intervention Delivery

The course was delivered twice at the District Police headquarters. The first course from 19–21 May 2019 and the second from 22–24 May 2019. Two courses enabled a greater proportion of officers to be released to attend training. The training was conducted by experienced local and national first aid trainers from the NRCS.

At the commencement of the program delivery, a 20-question test, routinely used by the NRCS, was used to explore participant’s first aid knowledge, and 5 questions, utilizing a 5-point Likert scale were used to assess their confidence in first aid application (Supplementary File S3). Knowledge questions covered for example, the purpose of first aid, airway opening, cardiopulmonary resuscitation, managing bleeding, the signs of shock, priorities in mass-casualty incidents and triage.

Immediately after completion of the training, the knowledge test and confidence scale measure were repeated, together with an evaluation seeking feedback on delivery of the training program.

A fully-packed, trauma response backpack was provided to be carried in each of the three district traffic police vehicles. The trainers also distributed incident report forms to all course participants to be completed after each road injury patient encountered for the six-month period of post-training follow-up (Supplementary File S4).

### 2.3. Phase 3: Follow-Up

Six months after the training, each participant was invited to take part in a follow up face-to-face verbal survey, to assess the extent to which the participants had incorporated first response skills gained from the training into practice (Supplementary File S5). The participants also completed the knowledge test to assess retention of course information and the confidence measure to assess self-belief in their abilities. Incident report forms were collected from the police stations. To assess the potential costs of providing first response training to all traffic police in Nepal, this study measured resources used, and the costs incurred of all aspects of the first-responder training program including training materials, equipment, and training personnel.

### 2.4. Data Analysis

To score the knowledge assessments at pre-test, post-test and follow up, five marks were allocated for correct answers to each of the 20 questions, giving a total score out of 100. Individual traffic officer’s scores were pooled for the cohort of police officers, so that no individual officer would be at risk of being penalized for their level of knowledge (29 participants at pre-test and post-test and 27 participants at follow up). A mean score at each time-point, together with a median, maximum and minimum score were calculated. All data analyses were conducted in MS Excel.

## 3. Results

### 3.1. Training Needs Assessment

Thirty-nine traffic police officers were stationed in the district, and all completed the training needs assessment. Thirty-eight (97%) of the thirty-nine agreed that first-aid was part of their responsibilities.

#### 3.1.1. First Aid Training and Equipment

Two-thirds of the participants (*n* = 25; 64.1%) said they had received no training in first-aid. Of those that had received the training (*n* = 14; 35.9%), the median (IQR) time since they had last received the training was 3 (1, 9) years. The median (IQR) length of the training was 8 (4, 24) h. The first aid training was provided by six different organizations outlined in Table 1.

Of the 14 participants who had been trained in applying first aid, 7 (50%) said they had no access to first-aid equipment at the time of the survey. Of those that had access to a first aid pack, the equipment mainly included wound dressings and triangular bandages.

#### 3.1.2. Experience of First Aid and Transporting the Injured

**Figure 1:**
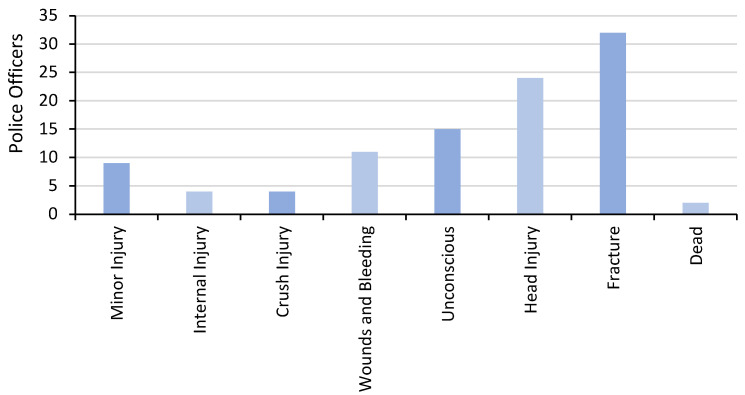
Road Traffic Injuries Transported to Hospital by Traffic Police in the Study District.

Of the 14 trained in first aid, 7 (50%) had applied first aid in the previous 12 months. In that time period, first aid skills were applied a median (IQR) frequency of 50 (3, 99) times per participant. Of the 39 participants, 37 (95%) had experience of transporting RTI victims to hospital with a wide range of injuries (Figure 1).

The participants who had been trained in first-aid and who had access to some first aid equipment (*n* = 7) were asked about what difficulties they faced when doing first aid at the scene of a road traffic collision (RTC). Three key issues were described: (1) lack of equipment; (2) lack of an ambulance for transport; (3) fear of criticism from the public when giving care.

### 3.2. Knowledge and Confidence

Twenty-nine participants were released from operational duties to attend the course. Knowledge of first aid prior to receiving the course was low, with a mean score for all participants of 35.5%. Confidence levels in applying first aid across a range of skills in practice was also low, with only 32.6% of participants reporting they felt ‘confident’ or ‘very confident’ across a range of skills. Immediately after completion of the three days of training, knowledge scores more than doubled to a mean of 74.9%, with improved results across all subject areas. Confidence levels also showed an improvement, with an average of 88%; an improvement of 45% from the baseline.

Twenty-seven of 29 participants (93%) were surveyed at 6 months. Two traffic police officers were lost to follow-up as they had been transferred to general police duties. At 6 months post-delivery, the mean first aid knowledge score was 57.1%, a drop of 17.8% from post-course (Table 2) and feeling ‘confident’ or ‘very confident’ was reported at 67%; a drop of 21% from post-course (Table 3). None of the course participants felt that any of the course content should be dropped, although 41% (*n* = 7) felt that the course should be longer.

### 3.3. Experience of Applying First Aid Skills

**Figure 2:**
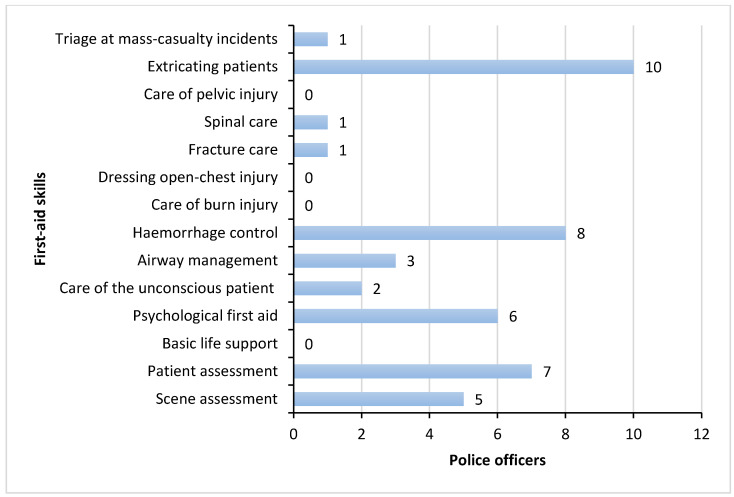
First Aid Skills Applied in Practice.

Participants reported they had attended a total of 303 RTCs where people had been injured during the six-month period of follow up. 12 participants (44.4%) stated that they had applied first aid to RTI victims on 92 occasions, with a median frequency of 2.5 (IQR 1, 8) times in that time period. 81% (*n* = 22) stated they had access to first-aid equipment. Figure 2 shows the first-aid skills applied in practice.

### 3.4. Perceived Barriers to Applying First-Aid

Issues that emerged when exploring barriers to providing first response care at the crash scene included that, if they were delayed in attending the crash scene, the patients had often already been transported to hospital by the public. They also reported experiencing pressure from the public to transfer the patient to hospital immediately and not deliver first aid, and on occasion they felt they lacked necessary equipment, such as a stretcher. Attending the crash scene presented them with competing police duties, as in addition to tending to the injured they needed to clear the road and record the crash event. Some reported a lack of encouragement from senior officers to deliver first aid.

### 3.5. Incident Report Forms

Only 4 incident report forms were collected in the 6-month data collection period, completed by three (11%) of the 27 staff available for follow up, despite first aid being reported to have been delivered on 92 occasions. A range of reasons for not completing the forms were given. Forgetting and time pressure were the most common. Lack of support and reinforcement from senior managers was also cited, along with assuming that more senior staff would complete the documentation.

### 3.6. Training Costs

The cost of the training course (including the 3 trauma packs) was £3682.87 (£127 per trainee). A summary of the first responder training course costs is listed in Table 4:

## 4. Discussion

Nepal is a country with a heavy burden of mortality and morbidity from road traffic injuries. The training needs assessment showed that traffic police were attending road traffic crashes and transporting casualties with limited or no standardized first-aid training and limited emergency resources. This is not uncommon in LMICs [16,17,18,19,20]. Previous studies have shown that where casualties are treated and/or rushed to hospital by untrained police or bystanders, they have much poorer outcomes and are more likely to die than if looked after by emergency medical personnel or trained first responders [20,21,22]. Published evidence on the training of traffic police in other settings has found it is not only feasible, but, when applied correctly, first-aid treatment for trauma victims has been reported to show significantly reduced mortality and morbidity [23,24].

The study team were able to develop and design a first-responder course for the traffic police, focused on managing traumatic injuries at RTCs. The course participants increased their knowledge of first aid and their confidence in applying those skills. They then went on to frequently perform first aid at the crash scene.

Knowledge of first aid and confidence in applying those skills in practice dropped significantly at 6 months, but not to baseline levels. At the follow up, some of the participants in our study identified the need for refresher training and indeed, this is an important part of retaining knowledge and confidence. Previous research conducted with lay first-aiders in Nepal has found that retention of first-aid skills reduces significantly over time and concluded that first-aid skills should be refreshed annually [25]. Other studies have shown increased retention of knowledge and self-assessed confidence in refresher training at 6 months [26,27]. The frequency of retraining must be balanced between the need to maintain skills and practical issues such as time out of work and financial costs.

Feedback on the training experience of traffic officers in this study showed the majority of the participants rated the training’s overall value as either ‘excellent’ or ‘good’. Delivery of the training needed to be flexible with regard to timing and duration so as to meet with the other competing duties of the traffic police.

The training delivery costs were modest and suggest it may be feasible to roll out to other districts with further investment. All items in the trauma pack (Supplementary File S6) were low cost and could be easily obtained locally. There is limited reporting on implementation and resourcing costs associated with first-responder training making it difficult to compare our findings with those of similar studies. A study in Uganda in 2008 reported the cost of a 1-day, basic first aid course for lay people, including the police, was approximately US$27 per participant [19]. The estimated cost of extending the training to other districts in Nepal compares favorably with the significant direct costs of road traffic injuries in Nepal [28] and costs per participant may reduce if the training was delivered to a larger cohort of officers.

Out of 303 RTC’s where patients have been injured, only 12 officers out of 27 applied their learning from the course. A number of barriers to the application of first-responder skills to RTI victims were identified. Delays in the police being dispatched and arriving on scene were identified by participants as factors resulting in the RTI victim having already been removed from the scene prior to their arrival. The lack of an identifiable, national, 3-digit phone number to summon the emergency services promptly may be a contributory factor to delays. Walker et al. [5] have reported some success with the introduction of the ‘102′ number in Kathmandu and other regions. As the public are frequently on scene before the police, having trained community first responders also makes intuitive sense. Research in other LMICs has shown this is feasible and can make a difference [17,22,23]. Barriers and facilitators to providing first aid to traffic injury victims is an area worthy of further study.

Having arrived on scene, although participants were able to frequently apply their skills for patient care, they sometimes felt pressured by the public to prioritize transferring the patient to hospital. There was a lack of public recognition that they were trained first responders, and that providing first aid may be more beneficial than immediate transportation. Possible solutions to this problem would be wearing an identifiable first-responder badge on their uniform and, if expanded nationally, a publicity campaign to inform the general public of their additional role.

Strengths of this study include that the course was designed after the training needs assessment, the engagement of the traffic police and Nepal Red Cross Society in the development and delivery of the intervention, and the subsequent follow up of participants at 6 months. Asking participants to recall the frequency of using first-aid skills is at risk of under- or over-reporting due to recall bias and is a significant study limitation. The limited number of contemporaneous incident report forms has meant a reliance on self-reporting and without the forms, it is not possible to determine the validity of the responses.

## 5. Conclusions

This study has demonstrated the feasibility of delivering a post-crash first-responder training course for the traffic police in Nepal, and the potential of such a program to improve outcomes in crash victims in the absence of formalized emergency medical services. Future studies should use mechanisms to capture the application of first-aid skills at the scene to supplement participant recall. The recognized barriers to providing first aid, including legal protection for first aiders, will also require further research. Reduced knowledge scores at 6 months and participant feedback, indicate the need to explore both the content, and potential frequency of refresher training. The cost of the three-day first-responder training course was minimal and the estimated cost of extending the training would compare favorably with the significant burden of the direct costs of road traffic injuries in Nepal. The study has identified the parameters for a future study of the clinical effectiveness and cost effectiveness of such a program which would be necessary prior to further roll-out of the program.

## Figures and Tables

**Table 1:** Organizations Providing First-Aid Training to the Traffic Police in the Study District.

First Aid Training Organisation	Number Trained (*n* = 14)
*Police (in-house) Trainer*	5
*Nepal Red Cross Society*	3
*Other provider:*	6
*Acute Private Hospital 1*	3
*Acute Private Hospital 2*	1
*Acute Private Hospital 3*	1
*Don’t Know*	1

**Table 2:** First Aid Course Knowledge Scores.

Variables	*n*	Median	Mean	Minimum Score	Maximum Score	Standard Deviation (SD)
Pre-test score	29	33.32	35.51	14.16	59.98	10.98
Post-test score	29	73.30	74.86	54.14	96.64	10.79
Six-month score	27	54.98	57.05	30.00	89.96	14.08

**Table 3:** First-Aid Confidence Levels.

	Confidence in:	Extremely Unconfident	Not Confident	Neutral	Confident	Very Confident
**Pre-First Responder Course**	*1. Performing CPR? (n = 29)*	5	4	14	5	1
	*2. Using dressings and tourniquets? (n = 28)*	6	6	7	8	1
	*3. Moving and handling patients? (n = 28)*	0	2	10	11	5
	*4. Managing broken bones? (n = 29)*	5	8	9	5	2
	*5. Using the recovery position? (n = 29)*	4	7	10	8	0
**Post-First Responder Course**	*1. Performing CPR? (n = 29)*	0	0	3	9	17
	*2. Using dressings and tourniquets? (n = 29)*	1	1	2	10	15
	*3. Moving and handling patients? (n = 29)*	0	0	1	8	20
	*4. Managing broken bones? (n = 29)*	0	1	4	8	16
	*5. Using the recovery position? (n = 29)*	1	1	3	8	16
**At 6 months follow-up**	*1. Performing CPR? (n = 27)*	0	0	11	7	9
	*2. Using dressings and tourniquets? (n = 27)*	0	0	11	7	9
	*3. Moving and handling patients? (n = 27)*	0	0	1	11	15
	*4. Managing broken bones? (n = 27)*	0	4	11	9	3
	*5. Using the recovery position? (n = 27)*	0	0	4	8	15

**Table 4:** First responder training program costs.

Activity Items	Quantity	Total (£)
*Trainee travel allowances*	29	328.41
*Trainer’s wages*	5	314.44
*Transportation*	2	87.15
*Food and accommodation costs for trainees and trainers*	34	1110.70
*Training materials*		
*Stationery and certificate*	29	95.66
*Gloves and mask*	29	33.39
*Pocket resuscitation face mask*	4	9.36
*Vinyl disposable bag valve mask (adult)*	4	42.96
*Cardiopulmonary resuscitation manikins*	4	775.20
*Trauma pack*	3	885.60
***Total***		**3682.87**

## Data Availability

The data presented in this study are available on reasonable request from the corresponding author. The data are not publicly available due to retaining the anonymity of participants.

## Supplementary Materials

The following supporting information can be downloaded at: https://www.mdpi.com/article/10.3390/ijerph19148481/s1. File S1: Training needs analysis; File S2: First responder course curriculum; File S3: Pre and post training questionnaire; File S4: Post incident patient report form; File S5: Follow up interview; File S6: Trauma pack contents.
