# Supplementary material for: Post-Crash First Response by Traffic Police in Nepal: A Feasibility Study
Source: Int J Environ Res Public Health. 2022 Jul 11;19(14):8481. doi: 10.3390/ijerph19148481 (PMC9323792; doi:10.3390/ijerph19148481)
Supplement: Supplementary file 1 [file ijerph-19-08481-s001.zip › Supplementary File S1 Training Needs Analysis.pdf]

Supplementary File 1. First responder training need assessment questionnaire for Makwanpur Traffic Police

1. Have you ever received first aid training?
   a. Yes
   b. No *(Skip to Q14)*

2. If yes, when was the last time you received the training? ___ days ___ months ___ years ago.

3. What was the name of the training? ____
____

4. Who provided the training?
   a. In house trainer (from police department)
   b. Nepal Red Cross Society
   c. Others *(specify______________________________________)*

5. What was the duration of the training? ___ hours ___ days ___ weeks

6. What first aid equipment/kits (items) was provided for your use after the training?
   a. Wound dressings
   b. Burns dressings
   c. Cervical collars
   d. Splints
   e. Triangular bandages
   f. Chest seals
   g. Tourniquets
   h. Stretchers
   i. Automatic external defibrillators
   j. Set of first aid kit
   k. Other *(specify_________________________________)*

7. Do you provide first aid to injured persons?
   a. Yes
   b. No *(Skip to Q14)*

8. How many times have you applied first aid in the last 12 months? *(Write numbers)* ____________

9. Level of knowledge

	Strongly disagree	Disagree	Neutral	Agree	Strongly agree
I feel that I know enough about different types of rescue.	1	2	3	4	5
I feel I know how to care for unconscious road injury victims.	1	2	3	4	5
I feel that I know the causes of a blocked airway in road traffic victims.	1	2	3	4	5
I feel that I know enough about catastrophic haemorrhage.	1	2	3	4	5
I feel that I know enough about injuries to bones, joints, tendons and ligaments.	1	2	3	4	5
I feel that I know how to extricate road injury victims and safe handling techniques.	1	2	3	4	5
I feel that I know enough about injuries to the head, neck, spine and chest.	1	2	3	4	5

10. Level of confidence

	Strongly disagree	Disagree	Neutral	Agree	Strongly agree
I feel that I can recognise the types of rescue; immediate, rapid and delayed.	1	2	3	4	5
I feel that I can assess the patient's level of response and place an unresponsive patient in to the recovery position.	1	2	3	4	5
I feel that I can recognise the cause of a blocked airway.	1	2	3	4	5
I feel that I can identify and control catastrophic haemorrhage using direct pressure.	1	2	3	4	5

I feel that I can recognise and immobilise a suspected fractured limb and joint dislocation.	1	2	3	4	5
I feel that I can apply a range of safe moving and handling techniques for road traffic injury victims.	1	2	3	4	5
I feel that I can recognise and administer first aid to a casualty with suspected head, spinal, chest injuries.	1	2	3	4	5

11. Do you do any first aid on your own or do you have anyone supporting you?

a. On my own *(Skip to Q13)*
b. Have someone supporting

12. If you have someone supporting, who is s/he?

____
____

13. Do you have access to any first aid equipment now?

a. Yes
b. No *(Skip to Q15)*

14. If yes, what are the first aid equipment and where are they?

____
____

15. What were the difficulties you faced when you were doing first aid at the scene?

____
____

16. Do you have experience of transporting injured or ill persons?

a. Yes
b. No

17. If yes, what types of injuries?

____
____

18. Do you think first aid should be your responsibility?

a. Yes
b. No

19. If no, why this is so? ________________________________
____

End
