# Supplementary material for: Post-Crash First Response by Traffic Police in Nepal: A Feasibility Study
Source: Int J Environ Res Public Health. 2022 Jul 11;19(14):8481. doi: 10.3390/ijerph19148481 (PMC9323792; doi:10.3390/ijerph19148481)
Supplement: Supplementary file 1 [file ijerph-19-08481-s001.zip › Supplementary File S2 First Responder Course Curriculum.pdf]

Supplementary File 2:. First Responder Training Programme

DAY 1 (8 hours)					
**Area of emphasis**	**Major components**	**Learning outcomes**	**Est. time**		**Equipment needed**
			Theory	Practical	
1. Principles of first aid	• Introduction and context<br>• Ethos and principles of humanitarian care of the sick and injured	That the participant should be able to: -<br>1.1 Identify what is first-aid and what is first response.<br>1.2 Recognise why first-aid is important.<br>1.3 Identify the principles of humanitarian care.<br>1.4 Identify the role and responsibilities of the first responder.<br>1.5 Recognise the legal obligations when administering first-aid.	50 min	0 min	• First aid manual x 1 per participant.
			Theory	Practical	
2. Scene assessment and the prehospital environment	• Personal safety<br>• Minimise cross-infection<br>• Mechanisms of injury<br>• Rescue	That the participant should be able to: -<br>2.1 Conduct a scene survey.<br>2.2 Identify the hazards at the scene of an incident.<br>2.3 Conduct a dynamic risk assessment.<br>2.4 Minimise the risk of cross-infection.<br>2.41 How to wash hands?<br>2.42 How to wear gloves?<br>2.5 Recognise the types of rescue; immediate, rapid and delayed.	45 min	20 min	• Disposable gloves x pair per participant
			Theory	Practical	
3. Patient assessment	• The primary survey<br>• Catastrophic haemorrhage<br>• Response<br>• Airway<br>• Breathing<br>• Circulation<br>• The secondary survey<br>• Vital signs<br>• Head to toe assessment	That the participant should be able to: -<br>*3.1* Recognise the importance of communication skills when dealing with a casualty.<br>*3.2* Conduct a primary survey using a DRCABC approach.<br>3.3 Identify catastrophic haemorrhage.<br>3.4 Control catastrophic haemorrhage by the use of direct or indirect pressure.<br>3.5 Open an airway and assess for breathing.<br>3.6 Check the circulation by checking the pulse.<br>3.7 Perform a head –to-toe assessment for further injury.	45 min	60 min	• First aid dressings x 2 per participant.<br>• Tourniquets x 1 per two participants.

4. Basic life support and automated external defibrillation	• Resuscitation for:<br>○ Infant<br>○ Child<br>○ Adult	That the participant should be able to: -<br>4.1 Demonstrate how to perform basic life support cardiopulmonary resuscitation (CPR) on an adult.<br>4.2 Demonstrate how to perform basic life support cardiopulmonary resuscitation (CPR) on a child.<br>4.3 Demonstrate how to perform basic life support cardiopulmonary resuscitation (CPR) on an infant.<br>4.4 Demonstrate how to use an automated external defibrillator.	Theory<br><br>30 min	Practical<br><br>90 min	• BLS Adult manikin x 1 per four participants.<br>• BLS Child manikin x 1 per four participants.<br>• BLS Infant manikin x 1 per four participants.<br>• BVM apparatus x 1 per manikin<br>• AED Training device x 1 per class

DAY 2 (8 hours)					
**Area of emphasis**	**Major components**	**Learning outcomes**	**Est. time**		**Equipment needed**
			Theory	Practical	
5. Psychological first aid	• How to prepare yourself mentally for first response	That the participant should be able to: -<br>5.1 Recognise the psychological and social needs of victims of injury.<br>5.2 Recognise common reactions to extreme stress including anxiety and fear.<br>5.3 Know that by witnessing traumatic events, first responders are also at risk of developing stress reactions and post-traumatic stress disorder.	60 min		
			Theory	Practical	
6. Care of the unconscious patient	• The recovery position	That the participant should be able to: -<br>6.1 Assess the patient's level of response.<br>6.2 Place an unresponsive patient in to the recovery position.	15 min	45 min	
			Theory	Practical	
7. Airway and breathing	• Causes of blocked airway<br>• Opening & maintaining a clear airway<br>• Basic airway adjuncts	That the participant should be able to: -<br>7.1 Recognise the causes of a blocked airway.<br>7.2 Clearing a blocked airway.	15 min	45 min	• Choking manikin x 1
			Theory	Practical	
8. Circulation and shock	• Recognising and initial care of wounds<br>• Animal bites<br>○ Snake<br>○ Dog<br>• Haemorrhage control<br>• Dressings<br>• Tourniquets	That the participant should be able to: -<br>8.1 Perform a wound assessment.<br>8.2 Recognise the type of bleeding; arterial, venous and capillary.<br>8.3 Apply a first aid dressing to an open wound.<br>8.4 Recognise the signs and symptoms of shock.<br>8.5 Apply a first aid dressing around impaled objects.<br>8.6 Assess a snake or dog bite and recognise the correct treatment pathway.	15 min	45 min	• First aid dressings x 1 per participant.<br>• Triangular bandages x 1 per participant.

9. Burn injuries	• Recognition & initial care of burn injuries	That the participant should be able to: -<br>9.1 Cool and dress a burn injury.	Theory<br>15 min	Practical<br>15 min	• First aid dressings x 1 per participant
10. Chest injuries	• Recognition & initial care of injuries to the chest	That the participant should be able to: -<br>10.3 Administer first aid to a casualty with suspected chest injuries.<br>10.4 Apply an occlusive dressing to an open chest wound.	Theory<br>15 min	Practical<br>45 min	• Occlusive chest seal dressings x 1 per four participants.

DAY 3 (8 hours)					
**Area of emphasis**	**Major components**	**Learning outcomes**	**Est. time**		**Equipment needed**
11.	• Recognition & initial care of injuries to bones, joints, tendons & ligaments	That the participant should be able to: -<br>11.1 Recognise and immobilise a suspected fractured limb.<br>11.2 Recognise and immobilise a suspected joint dislocation.	Theory<br>15min	Practical<br>45 min	• Triangular bandages x 1 per participant.<br>• SAM splints x 1 per two participants.
12.	• Recognition & initial care of injuries to the head, neck and spine	That the participant should be able to: -<br>12.1 Administer first aid to a casualty with suspected head and spinal injuries.<br>12.2 Administer a log-roll to a patient with a spinal injury.<br>12.3 Apply a cervical collar to a patient with a suspected neck injury.	Theory<br>15 min	Practical<br>45 min	• Triangular bandages x 1 per participant.<br>• Cervical collars x 1 per four participants.
13.	• Recognition and initial care of pelvic injuries.	That the participant should be able to: -<br>13.1 Administer first aid to a casualty with suspected pelvic injury.	Theory<br>15 min	Practical<br>45 min	

					• Triangular bandages x 1 per participant.
14. Moving and handling patients	• Extricating patients<br>• Safe handling techniques<br>• Improvised equipment	That the participant should be able to: -<br>14.1 Apply a range of safe patient moving and handling techniques.<br>14.2 Demonstrate improvised skills and equipment when moving patients.<br>14.3 Demonstrate how to safely remove a patient from a car crash.<br>14.4 Plan how to evacuate patients from the incident scene whilst maintaining neutral alignment of the spine.<br>14.5 Demonstrate how to improvise a stretcher and package a patient for transport to hospital.	Theory<br>15 min	Practical<br>120 min	• Cervical collars x 1 per four participants.<br>• 1m x 2.5m tarpaulin x 1 per four participants.<br>• 1m x 2.5m blanket x 1 per four participants.<br>• 2 x poles per four participants.<br>• 1 x canvas stretcher/scoop stretcher per four participants.
15. Triage at mass-casualty incidents	• Triage system with multiple casualties	That the participant should be able to: -<br>15.1 Demonstrate how to triage in road crashes with more than one casualty.	Theory<br>30 min	Practical	
16. First aid equipment and report forms	• Equipment<br>• Reporting	That the participant should be able to: -<br>16.1 Demonstrate how to complete a Post-incident Patient Report Form.<br>16.2 Identify the range of equipment supplied in the first response kit.	Theory<br>40 min	Practical	• First responder kit for demonstration purposes.<br>• Copies of blank patient report records.

The curriculum utilises 'best practices' and concepts gathered from established courses, including first-aid/responder, wilderness and combat medicine; the World Health Organization, International Liaison Committee on Resuscitation and the international Federation of Red Cross and red Crescent Societies. The program stresses safety first, emphasises the risk/benefit considerations of treatments and evacuation practices, and suggests the use of improvised skills and equipment.

Human resources

Total number of participants: 39

Total number of training sites: 1

Total number of participants per session: To be agreed with Traffic Police

Total number of trainers per site: 4
