# Supplementary material for: Post-Crash First Response by Traffic Police in Nepal: A Feasibility Study
Source: Int J Environ Res Public Health. 2022 Jul 11;19(14):8481. doi: 10.3390/ijerph19148481 (PMC9323792; doi:10.3390/ijerph19148481)
Supplement: Supplementary file 1 [file ijerph-19-08481-s001.zip › Supplementary File S3 Pre and post training questionnaire.pdf]

Supplementary File 3: Pre and post-training questionnaire

Please circle the correct answers from the given options. Participants may need to circle multiples answers if needed.

First Responder's Name: ___________________________

SECTION 1: KNOWLEDGE RELATED QUESTIONS
**1. As per the pillars mentioned in the Nepal Road Safety Action Plan, which pillars mention the roles of volunteers in first aid and road safety?**<br>a. Road Safety Management<br>b. Safer roads and mobility<br>c. Safer vehicles<br>d. **Safer road users**<br>e. **Post-crash response**
**2. Why is first aid important in road crashes?**<br>a. To examine the injured person<br>b. To ensure safety in crash site<br>c. **To save life of injured person**<br>d. To bring changes in the health of communities through medical treatment<br>e. None of the above
**3. How do you support injured person in road crashes?**<br>a. Provide safety in crash site, provide first aid to injured person, transfer injured person, check the vital signs of injured person<br>b. **Provide safety in crash site, check the vital signs of injured person, provide first aid to injured person, transfer the injured person**<br>c. Provide first aid to injured person, check the vital signs of injured person, provide safety in crash site, transfer the injured person<br>d. Check the vital signs of injured person, provide first aid to injured person, transfer the injured person, provide safety in crash site<br>e. None of the above
**4. Which is not included in patient assessment?**<br>a. Scene assessment<br>b. Check for dangers<br>c. Check airway, breathing and circulation<br>d. **Immediately taking to hospital**
**5. How can you open the airway?**<br>a. Opening mouth<br>b. Cleaning nose<br>c. **Head tilt and chin lift**<br>d. Ask to cough
**6. How can you check a person is breathing?**<br>a. Ask people to breathe<br>b. Ask people to blow<br>c. As people to cough<br>d. **Look, listen and feel for breathing**
**7. When doing CPR, what is the ratio of rescue breathing and chest compressions for the adult?**<br>a. 20:3<br>b. 30:3<br>c. **30:2**<br>d. Don't know
**8. Which of the following is the correct order to provide first aid to an injured person with severe bleeding?**<br>a. Keep injured person in a comfortable position, raise the body part that is bleeding, press, tie bandage, treatment of shock, take the injured to hospital<br>b. Raise the body part that is bleeding, keep injured person in a comfortable position, press, tie bandage, treatment of shock, take the injured to hospital<br>c. **Press the bleeding part, keep injured person in a comfortable position, apply dressing, treatment of shock, take the injured to hospital**<br>d. Treatment of shock, press the bleeding part, keep injured person in a comfortable position, raise the body part that is bleeding, apply pressure indirectly, tie bandage, take the injured to hospital<br>e. None of the above
**9. What components need to apply when providing psychological first aid?**<br>a. Preparation

b. Observation
c. Active listening
d. Linkage
e. **All of the above**

10. Which of the following indicate a spinal injury?
a. Back swelling in the midline of the back
b. Back pain in the midline of the back
c. Loss of feeling or movement in the legs
d. **All of the above**

11. What complications will occur when a person with head and spinal injury is not treated on time?
a. Probability of hand fracture
b. **Partial paralysis may occur**
c. **Complete paralysis**
d. Severe bleeding might occur
e. None of the above

12. What are the symptoms of the muscle injury?
a. **Pain**
b. **Swelling**
c. **Hard to walk**
d. Loss of feeling or movement in the limbs

13. Which of the following are the signs and symptoms of shock?
a. **Pale face**
b. **Irregular pulse rate**
c. **Sweating**
d. Fever
e. None of the above

14. What should not you do with a patient in shock?
a. Lay them down
b. **Raise their legs**
c. Keep them warm, cover with a blanket
d. **Give them to eat and drink**

15. Which of the following is a sign of a broken bone?
a. Pain and swelling
b. Loss of movement
c. Irregular angulation of the limb
d. **All of the above**

16. When would you apply a tourniquet?
a. **When a limb has become amputated or heavy arterial bleeding if not controlled by direct pressure**
b. With small cuts and grazes
c. With burns
d. With snake bites

17. Which triage tag do you use to correctly categorise injured person who has catastrophic bleeding from his/her hand?
a. Green tag
b. **Red tag**
c. Green tag first and then red tag
d. Use both tags together
e. None of the above

SECTION 2: CONFIDENCE RELATED QUESTIONS

	Extremely unconfident	Not confident	Neutral	Confident	Very confident
**1.** How confident are you at performing CPR?	1	2	3	4	5
**2.** How confident are you with using dressings and tourniquets?	1	2	3	4	5
**3.** How confident are you at moving and handling patients?	1	2	3	4	5
**4.** How confident are you with managing broken bones?	1	2	3	4	5
**5.** How confident are you at putting a casualty in the recovery position?	1	2	3	4	5

End
