# Supplementary material for: Post-Crash First Response by Traffic Police in Nepal: A Feasibility Study
Source: Int J Environ Res Public Health. 2022 Jul 11;19(14):8481. doi: 10.3390/ijerph19148481 (PMC9323792; doi:10.3390/ijerph19148481)
Supplement: Supplementary file 1 [file ijerph-19-08481-s001.zip › Supplementary File S4 Post incident patient report form.pdf]

Supplementary File 4: Post-incident patient report form

1. First responder's ID/Name: _________________________

2. Today's Date: _______________ *(dd/mm/yyyy)*

3. Time of injury: ______________ *(am/pm)*

4. Time on scene: ______________ *(am/pm)*

5. Age category of injured person:
   a. Old person
   b. Adult
   c. Young person
   d. Child
   e. Baby

6. Gender:
   a. Male
   b. Female
   c. Others

7. Location of incident:
   a. _________________ Sub-metro/Rural/Municipality
   b. _________________ Ward No.
   c. _________________ Locality
   d. _________________ Highway/road

8. What injuries did the person have? *(Please circle injured areas after head to toe exam)*

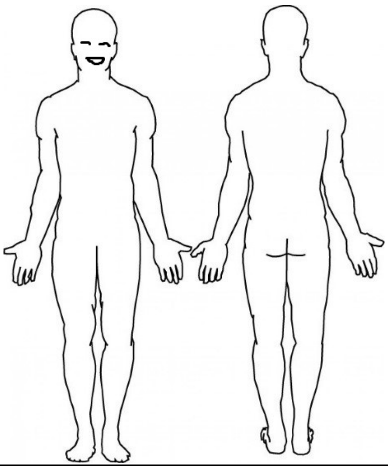

9. Assessment/Problem list:
   1. ____
   2. ____
   3. ____
   4. ____

10. Severity of injury:
    a. Fatal
    b. Severe
    c. Minor

11. What care did you provide? *(Please circle all actions taken)*
    a. Assessed ABCs
    b. Provide CPR
    c. Stopped bleeding
    d. Person turned on side
    e. Splinted fracture
    f. Other *(specify _________________________)*

12. Where was the casualty taken?
    a. Hetauda District Hospital
    b. Chure Hill Hospital
    c. Other *(specify ___________________)*

13. Did you transport the casualty?
    a. Yes *(skip to Q15.)*
    b. No

14. If you did not transport the casualty, who did?
    a. Relatives
    b. Bystanders
    c. Ambulance
    d. Other *(specify _________________________)*

15. What equipment did you use? *(Please circle all items that you used)*
    a. Gloves
    b. Splint
    c. Crepe bandage
    d. Wound dressings
    e. Burns dressings
    f. Splints
    g. Triangular bandages
    h. Chest seals
    i. Tourniquets
    j. Stretchers
    k. Other *(specify __________________)*

End
