# Supplementary material for: Post-Crash First Response by Traffic Police in Nepal: A Feasibility Study
Source: Int J Environ Res Public Health. 2022 Jul 11;19(14):8481. doi: 10.3390/ijerph19148481 (PMC9323792; doi:10.3390/ijerph19148481)
Supplement: Supplementary file 1 [file ijerph-19-08481-s001.zip › Supplementary File S5 Follow up interview.pdf]

Supplementary File 5: Follow-up interview questionnaire at six months

<u>Section 1: Basic information</u>

1.1 Are you completing post-incident patient report form?
- a. Yes
- b. No

1.2 How many road traffic <u>injuries</u> have you witnessed in Makwanpur District in the past six months?
(Write exact number) ___________________

<u>Section 2: Knowledge retention of first aid skills</u>

2.1 As per the pillars mentioned in the Nepal Road Safety Action Plan, which pillars mention the roles of volunteers in first aid and road safety?
- a. Road Safety Management
- b. Safer roads and mobility
- c. Safer vehicles
- **d. Safer road users**
- e. **Post-crash response**

2.2 Why is first aid important in road crashes?
- a. To examine the injured person
- b. To ensure safety in crash site
- **c. To save life of injured person**
- d. To bring changes in the health of communities through medical treatment
- e. None of the above

2.3 How do you support injured person in road crashes?
- a. Provide safety in crash site, provide first aid to injured person, transfer injured person, check the vital signs of injured person
- **b. Provide safety in crash site, check the vital signs of injured person, provide first aid to injured person, transfer the injured person**
- c. Provide first aid to injured person, check the vital signs of injured person, provide safety in crash site, transfer the injured person
- d. Check the vital signs of injured person, provide first aid to injured person, transfer the injured person, provide safety in crash site
- e. None of the above

2.4 Which of the following is not included in patient assessment?
- a. Scene assessment
- b. Check for dangers
- c. Check airway, breathing and circulation
- **d. Immediately taking to hospital**

2.5 How would you open the airway?
- a. Opening mouth
- b. Cleaning nose
- **c. Head tilt and chin lift**
- d. Ask to cough

2.6 How would you check a person is breathing?
- a. Ask people to breathe
- b. Ask people to blow
- c. As people to cough
- **d. Look, listen and feel for breathing**

2.7 When doing CPR, what is the ratio of rescue breathing and chest compressions for the adult?
- a. 20:3
- b. 30:3
- **c. 30:2**
- d. Don’t know

2.8 Which of the following is the correct order to provide first aid to an injured person with severe bleeding?
- a. Keep injured person in a comfortable position, raise the body part that is bleeding, press, tie bandage, treatment of shock, take the injured to hospital
- b. Raise the body part that is bleeding, keep injured person in a comfortable position, press, tie bandage, treatment of shock, take the injured to hospital

c. **Press the bleeding part, keep injured person in a comfortable position, apply dressing, treatment of shock, take the injured to hospital**
d. Treatment of shock, press the bleeding part, keep injured person in a comfortable position, raise the body part that is bleeding, apply pressure indirectly, tie bandage, take the injured to hospital
e. None of the above

2.9 What components need to apply when providing psychological first aid?
a. Preparation
b. Observation
c. Active listening
d. Linkage
e. All of the above

2.10 Which of the following indicate a spinal injury?
a. Back swelling in the midline of the back
b. Back pain in the midline of the back
c. Loss of feeling or movement in the legs
d. All of the above

2.11 What complications will occur when a person with head and spinal injury is not treated on time?
a. Probability of hand fracture
b. Partial paralysis may occur
c. Complete paralysis
d. Severe bleeding might occur
e. None of the above

2.12 What is symptom of a muscle injury?
a. Pain
b. Swelling
c. Hard to walk
d. Loss of feeling or movement in the limbs

2.13 Which of the following are the sign and symptoms of shock?
a. Pale face
b. Irregular pulse rate
c. Sweating
d. Fever
e. None of the above

2.14 What should not you do with a patient in shock?
a. Lay them down
b. Raise their legs
c. Keep them warm, cover with a blanket
d. Give them to eat and drink

2.15 Which of the following is a sign of a broken bone?
a. Pain and swelling
b. Loss of movement
c. Irregular angulation of the limb
d. All of the above

2.16 When would you apply a tourniquet?
a. When a limb has become amputated or heavy arterial bleeding if not controlled by direct pressure
b. With small cuts and grazes
c. With burns
d. With snake bites

2.17 Which triage tag do you use to correctly categorised injured person who has catastrophic bleeding from his/her hand?
a. Green tag
b. Red tag
c. Green tag first and then red tag
d. Use both tags together
e. None of the above

Section 3: Level of confidence in applying first aid skills

3.1 Do you do any first aid on your own or do you have anyone supporting you?
  a. On my own
  b. Have someone supporting

	**Extremely unconfident**	**Not confident**	**Neutral**	**Confident**	**Very confident**
3.2 How confident are you at performing CPR?	1	2	3	4	5
3.3 How confident are you with using dressings and tourniquets?	1	2	3	4	5
3.4 How confident are you at moving and handling patients?	1	2	3	4	5
3.5 How confident are you with managing broken bones?	1	2	3	4	5
3.6 How confident are you at putting a casualty in the recovery position?	1	2	3	4	5

Section 4: Self-reported use of any first aid and any equipment used

4.1 Did you provide any first aid in the previous 6 months using skills learned from the training?
  a. Yes
  b. No *(Skip to Q4.4)*

4.2 If yes, what first aid you provided? *(Select all that apply)*
  a. Scene assessment
  b. Patient assessment
  c. Basic life support
  d. Psychological first aid
  e. Care of the unconscious patient
  f. Opening and maintaining a clear airway/ basic airway adjuncts
  g. Haemorrhage control
  h. Care of burn injury
  i. Apply an occlusive dressing to an open chest and neck wound
  j. Immobilise fractured limb and joint dislocation
  k. Apply a log-roll to a patient with a spinal injury
  l. Apply three padded triangular bandage or blanket for the suspected pelvic injuries
  m. Extricating patients
  n. Triage at mass-casualty incidents

4.3 How many times have you applied first aid in the past 6 months? *(Write numbers)* ______________

4.4 Do you have access to any first aid equipment now?
  a. Yes
  b. No

Section 5: Reflection on the first aid training programme provided

5.1 Do you think first aid should be your responsibility?
  a. Yes
  b. No

5.2 What do you think should be added to this training?
____
____

5.3 What do you think should be dropped from this training?
____
____

Section 6: Any perceived barriers to applying first aid skills

6.1 What were the difficulties you faced when you were doing first response at the scene?
____
____

End
