# Supplementary material for: Post-Crash First Response by Traffic Police in Nepal: A Feasibility Study
Source: Int J Environ Res Public Health. 2022 Jul 11;19(14):8481. doi: 10.3390/ijerph19148481 (PMC9323792; doi:10.3390/ijerph19148481)
Supplement: Supplementary file 1 [file ijerph-19-08481-s001.zip › Supplementary File S6 Trauma Pack Contents.pdf]

# Supplementary File 6: Trauma Pack Contents

| | |
|---|---|
| 1 x Dixie Ultimate Pro Backpack - Navy Blue; | 2 x Europlast Fabric Tape Roll - 2.5cm x 4.5m; |
| 2 x Quality Cotton Crepe Bandage - 10cm; | 1 x Transpore Allergy Tape - 2.5cm; |
| 4 x Sterile Calico Triangular Bandage - 90x127cm; | 2 x Jelonet 10cm x 10cm Paraffin Gauze - Pack 10; |
| 1 x Silver Foil Space Blanket – Adult; | 10 x Reliswab Non-Woven Sterile Swabs - 7.5 x 7.5cm - Pack of 5; |
| 1 x Sterile Burn Bag for Hands and Fingers; | 3 x T4 Trauma Wound Dressing Pad - 10cm x 18cm; |
| 1 x BurnKling 100m Film; | 3 x T6 Trauma Wound Dressing Pad - 15cm x 18cm; |
| 1 x Ambu Perfit Ace Extrication Collar – Adjustable; | 2 x TraumaFix Military Dressing - 20cm x 19cm; |
| 1 x Sterile Eye Wash Bottle - 250ml; | 3 x High Absorbency Multi Trauma Dressing; |
| 2 x No 1 Ambulance Dressing; | 1 x Community Sharpsafe Box - 8" / 0.6L; 2 x IV Solution Sterile Giving Set; |
| 2 x No 2 Ambulance Dressing; | 1 x TPAK Chest Decompression Needle; |
| 2 x No 3 Ambulance Dressing; | 1 x Asherman Chest Seal; |
| 2 x No 4 Ambulance Dressing; | 2 x Combat Application Tourniquet - C-A-T™ - Black; |
| 2 x Eye Pad Sterile HSE Dressing; | 1 set Guedel Disposable Airway; |
| 4 x SAM Splint Orange/Blue - 36" - 91.5cm x 11.5cm) | 1 x Tuf Cut Paramedic Shears – Red; |
